# “The Record is Our Work Tool!”—Physicians’ Framing of a Patient Portal in Sweden

**DOI:** 10.2196/jmir.5705

**Published:** 2016-06-27

**Authors:** Christiane Grünloh, Åsa Cajander, Gunilla Myreteg

**Affiliations:** ^1^ School of Computer Science and Communication KTH Royal Institute of Technology Stockholm Sweden; ^2^ Institute of Informatics TH Köln - University of Applied Sciences Gummersbach Germany; ^3^ Department of Information Technology Uppsala University Uppsala Sweden; ^4^ Örebro University School of Business Örebro University Örebro Sweden

**Keywords:** patient-accessible electronic health records, medical records, personal health records, eHealth services for patients, patient portal, technological frame, physicians

## Abstract

**Background:**

Uppsala County in Sweden launched an eHealth patient portal in 2012, which allows patients to access their medical records over the Internet. However, the launch of the portal was critically debated in the media. The professionals were strongly skeptical, and one reason was possible negative effects on their work environment. This study hence investigates the assumptions and perspectives of physicians to understand their framing of the patient portal in relation to their work environment.

**Objective:**

The study uses the concept of technological frames to examine how physicians in different specialties make sense of the patient portal in relation to their work environment.

**Methods:**

A total of 12 semistructured interviews were conducted with physicians from different specialties. Interviews were transcribed and translated. A theoretically informed thematic analysis was performed.

**Results:**

The thematic analysis revealed 4 main themes: work tool, process, workload, and control. Physicians perceive medical records as their work tool, written for communication within health care only. Considering effects on work environment, the physicians held a negative attitude and expected changes, which would affect their work processes in a negative way. Especially the fact that patients might read their test results before the physician was seen as possibly harmful for patients and as an interference with their established work practices. They expected the occurrence of misunderstandings and needs for additional explanations, which would consequently increase their workload. Other perceptions were that the portal would increase controlling and monitoring of physicians and increase or create a feeling of mistrust from patients. Regarding benefits for the patients, most of the physicians believe there is only little value in the patient portal and that patients would mostly be worried and misunderstand the information provided.

**Conclusions:**

Supported by the study, we conclude: (1) The transfer of a paper-based health care process where patients read on paper into a digital process challenges current work practices and has consequences for the work environment. Mostly, this is explained by the changing positions between the physicians and the patient: the latter can drive the process, which reduces the physicians’ ability to guide the patient. (2) The physicians’ experiences were expressed as worries: patients would not understand the content of the record and become unnecessarily anxious from misunderstandings. The concerns are to some extent based on a generalized view of patients, which might disregard those, who already actively participate in health care. This study hence reveals a need to provide physicians with information about the values for patients from using patient portals. (3) A change of work practices may be beneficial to increase patient participation, but such changes should preferably be designed and discussed with physicians. However, the strong resistance from the physicians made this challenging when launching the patient portal.

## Introduction

### Patient Portals and Aim of this Paper

The prospect of increasing costs in health care due to demographic changes and the increase of chronic diseases motivates politicians and policymakers to support patients to participate actively in their care. Enabling patients easy access to their medical record is one approach to make them active in managing their own health [1]. As previous paper-based medical records have been digitalized already, it may seem as a natural consequence that patients can access them over the Internet, for example, through secure eHealth services such as a patient portal. Especially, because in numerous countries, the right to access one’s medical record is constituted by law. However, the introduction of a patient portal is accompanied by major concerns, especially from health care professionals, who stress, for example, that Web-based access might lead to an increased workload and privacy risks [2]. Research has shown that such concerns discourage health care professionals from embracing the technology, and that for a patient portal to reach its full potential, patients and physicians need to see it as a technology that adds value to care [3]. Miller et al conclude furthermore that research is needed to examine ways how portals can be implemented to address providers’ concerns [3].

In Sweden, the introduction of a patient portal led to strong and mostly negative reactions from health care professionals, especially physicians and their trade union. This was shown in a Web survey from 2013, where 82% of the 385 physicians strongly disagreed or disagreed, that Web-based access to medical records is a good reform [4]. Moreover, the same survey also showed that the physicians were negative to giving the relatives of patients access to the medical records and were reluctant toward the eHealth service as such [5]. Another exploratory study has shown that physicians have different assumptions and perspectives that affect their use of technology and how they view patient empowerment [6]. However, so far, there are few qualitative studies on medical professionals’ views of patient portals.

The aim of this paper was to further the understanding of the perspectives of physicians in Uppsala when it comes to the patient portal and its effects on their work environment.

### Background and Theory

#### The Patient Portal

In Sweden, the patient portal was launched in Uppsala County Council to its 350,000 patients in 2012 as a part of a large European Union project. This launch was the result of 15 years of work in several projects and efforts that included law changes as well as lawsuits, as the development project ran into accusations of violating, for example, the Work Environment Act (1977:1160) [7]. The overarching aim of the patient portal is to contribute to patient empowerment and patient participation. The portal makes it possible for patients to log in on a Web service and read their health care information and test results and to use about 10 eHealth services. These services include, for example, booking appointments, following referrals, and reading a list of names of all health care professionals who have entered the medical record (so-called “log list”).

Two aspects of the patient portal are relevant to this study: (1) patients being able to read their records with or without delay and (2) the log list. The first aspect concerns patients’ access to their medical record with or without a delay. At the time of the interviews, only the signed medical notes and test results were shown to the patients. The patients could access their medical records either immediately after their physician had signed the note or if unsigned at the earliest after 14 days. This way of showing the information with a delay from when it was originally written is called a “respite”. This functionality has been changed in that the 2-week respite has been removed altogether. Thus, in the current version of the system used in Uppsala County, all records are accessible immediately to patients. The patient could choose after log-in, whether all or only the signed records will be at display. Most patients (98%) chose to see also the unsigned records [8], which would be specifically marked to distinguish them from the already signed notes.

The second aspect, the log list, came about due to patient integrity. The electronic medical record (EMR) is accessible to all public medical clinics in the county. The log list was implemented as a service that makes it possible for patients to look at a list of names of the health care providers who have logged on to their medical record. One underlying idea was that patients would easily recognize names of people familiar to them, such as a neighbor or a relative, who do not have legal permission to read the records. Patients already had the possibility to request a list of those who had read their record; this Web service is a means to simplify this process for the patients.

That citizens would have access to a portal with different patient services, including Web-based accessible medical records, was a controversial issue, and the reactions in media were strong with more than 70 posts in newspapers [7]. The concerns of the health care professionals in this media turbulence were mostly that patients would not be able to make use of the information provided and that medical records were intended for use by health care professionals only. However, concerns were also raised regarding the effect of the new eHealth services on the work environment in health care [7].

#### The Digital Work Environment in Health Care

The problems with information technology (IT) in health care in relation to usability have been described numerous times, for example, [9] and [10]. There is also research describing how the work environment of health care professionals is becoming more stressful due to factors such as irrelevant and unnecessary work [11] and a focus on efficiency and patient-centered care. However, according to a recent survey with staff in residential age care units in Sweden, higher levels of person-centered care was associated with higher levels of satisfaction with work and care [12].

The digitalization of previous paper-based documents may simplify work task to such an extent that the original purpose of the document may change. For example, the Medical Informatics Committee of the American College of Physicians outlines in a position paper how the clinical documentation process has developed over time and is now used for multiple other purposes than direct care of the patient [13]. This has led to requirements that influenced the format and content of the documentation [13]. In their policy recommendations, Kuhn et al state that the primary purpose of clinical documentation should be to “support patient care and improve clinical outcomes through enhanced communication” and that patient access to progress notes and medical records “may offer a way to improve both patient engagement and quality of care” [13].

#### Technological Frames

The term technological frames (TF) was coined by Orlikowski and Gash (1994) and concern the “assumptions, expectations, and knowledge” people use to understand the technology in their organization [14]. Technological frames in that sense do not only concern the role and nature of the technology but also its conditions, consequences, and applications. The artifact itself and the contexts of design and use are formative aspects of the TF [14]. The perception of a new technology can also be regarded as a social phenomenon, in that each individual is exposed to the attitudes of others, and these take part of the formation of the individual’s attitudes, beliefs, and values [15]. In the present research, the group of physicians is under investigation, based on the idea that this group is not homogenous: depending on their experiences, work environment, type of patients, and so forth, the TFs of physicians may vary [16].

Previous research has found that the idea of domains of TFs can be used to characterize the interpretations made by participants [14]. Orlikowski and Gash identified the 3 frame domains:

*Nature of Technology:* people’s images of the technology and their understanding of its capabilities and functionality.

*Technology Strategy:* understanding of the motivation or vision behind the adoption decision and its likely value to the organization.

*Technology in Use:* understanding of how the technology will be used on a day-to-day basis and the likely or actual conditions and consequences associated with such use.

The implementation of a new technology is often accompanied by skepticism and inertia due to different assessments of the value of the technology in use and the effects on the particular work environment. The framework of TFs is supposed to analyze, explain, and anticipate outcomes around the technological change in organizations [14]. Although frames can be facilitating in ambiguous situations in terms of reducing the uncertainty and providing a basis for taking action, they can also be constraining when they “reinforce unreflective reliance on established assumptions and knowledge, distort information to make it fit existing cognitive structures, and inhibit creative problem solving” [14].

In this study, to reach the aim to understand the physicians’ perspectives of the portal and its effects on their work environment, the frame domains from previous research were adopted.

## Methods

### Interview Content and Data Collection

Semistructured interviews were conducted in the summer of 2013, about 6 months after the patient portal was launched. A total of 12 physicians were interviewed by 3 different researchers. All researchers used the same template for the interviews to cover the required areas of interest. The template consisted of 27 questions (see Multimedia Appendix 1) and was developed in cooperation through a number of meetings. All interviews were done face to face except 1, which was carried out by email. The email interview was initially also planned to be conducted face to face but had to be rescheduled 3 times. After the last appointment had to be cancelled as well, the respondent suggested the possibility to answer via email, which was then accepted by the research team.

### Participants

The ambition should be to select respondents in a manner that helps the researcher to learn as much as possible and to find representative respondents [17]. To get access to physicians who were willing to take part in an interview proved to be a greater obstacle than was anticipated. Different strategies were applied to find physicians, for example, contacting heads of departments, mailing lists, and so forth, which makes it impossible to indicate the exact number of individuals who were asked to participate. However, all physicians who were willing to take part were interviewed.

The project succeeded in getting a positive response from physicians in 4 different specialties: orthopedics, oncology, emergency medicine, and internal medicine. The characteristics of the interviewed physicians (N=12) can be found in Table 1.

**Table 1:** An overview of the interviewees (N=12).

Characteristics		n (%) or mean (range)
Specialty, n		
	Orthopedics (Ortho)	5
	Oncology (Onco)	3
	Emergency Medicine (EM)	2
	Internal Medicine (IM)	2
Gender, n (%)		
	Female	5 (42)
	Male	7 (58)
Work experience in years, mean (range)		14 (2-30)

### Analysis

All interviews were transcribed, translated, and repeatedly read by all authors. The translation took place before the analysis due to the international composition of the research team. A thematic analysis was conducted [18] by using the Computer-Assisted Qualitative Data Analysis Software (CAQDAS) *Dedoose* [19]. This Web-based software was used with an additional layer of encryption to meet data privacy requirements and by this, it allowed the researchers to analyze and code the data both independently and jointly. After the familiarization with the data, initial codes were generated informed by the theory of TFs, but still allowing for an inductive approach. The first set of codes was: Concerns, Physicians Patient Relationship, Experience e-health Services, Implementation & Deployment, Medical Records Online, Opportunities, Patient Empowerment, Work Environment.

The Dedoose Training Center [19] was used early in the analysis to evaluate the coding agreement between researchers (intercoder reliability) and to engage in early discussions about codes and possible themes. The training session enabled a comparison between the coders. Joint coding sessions were carried out, in which the understanding of the different codes was discussed and differences in coding style were identified (eg, length of excerpt). During the joint coding, some codes and subcodes were added (Deliver Bad News, Authority, Expertise, Process Driver).

After the coding, all excerpts were exported, thoroughly read through repeatedly, and commented on. The excerpts were printed to facilitate collation, clustering, and the development of a thematic map. The clustered extracts were read again for each theme to review the internal homogeneity (cf. [18]). Part of the analysis process was also the iterative development of a thematic map and the description of the themes in writing, which helped to identify the relationship between the different themes. The quotes used in this paper have been slightly edited to be more readable.

## Results

**Figure 1:**
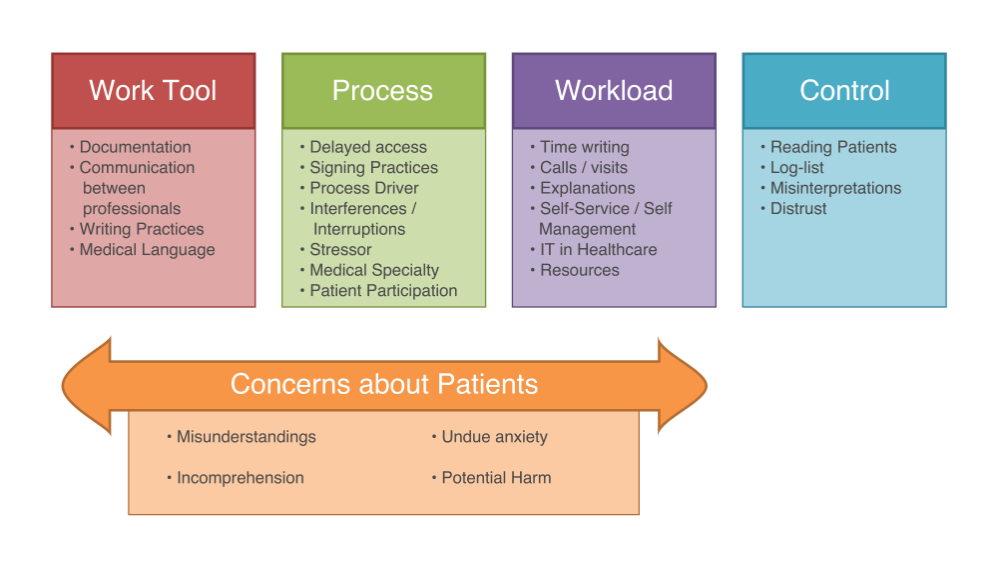
Identified themes from interviews with physicians.

The analysis revealed 4 themes, which are: Work Tool, Process, Workload, and Control (Figure 1). Related to the first 3 themes are the physicians’ concerns about patients (Figure 1), for example, patients misunderstanding or not comprehending the records; experiencing undue anxiety, and possibly being harmed by this. The concerns about patients are explicitly stated in relation to these 3 themes and thus presented here as a part of the respective theme instead of as a separate theme in itself.

As stated previously, domains of TFs are *nature of technology, technology strategy*, and *technology in use.* The identified themes in this study are instantiations of the domains of TFs, in that work tool refers to the *nature of technology*, process and workload refers to *technology in use*, and control refers to both, *technology in use* and *technology strategy.*

In the following sections, each of the 4 main themes will be described in detail.

### Work Tool

This theme concerns the physicians’ view of the EMR as a work tool, used for documentation and communication between health care professionals. Patients reading their medical records through the patient portal raised concerns, especially regarding the difficulties to understand the content due to the use of medical terms.

The medical records are written in such a language that I believe many patients have difficulty understanding the text at all.

Ortho-5

The record is our work tool; it has never been the patients’.

Ortho-1

The physicians discussed their work practices in how they write (eg, medical language, suspected diagnosis) and were concerned that the record as a work tool might lose its purpose if one would change the writing to make it more comprehensible for laypeople.

If you then are forced to use terms other than the ones you do now, then I do not think you get the same rigor as it has today in the record. Or it may be that many of the information that I might have included if the patient would not be reading the record I will not include now.

Ortho-4

It is not fair to me as a doctor to change my way of dictating, change my way to express myself for patients to understand. Then the information will be inaccurate, useless, do not fulfill its purpose, and rather be a risk I would say. And it is also a value, /.../ to bring a discussion about tentative diagnoses and stuff that is not perhaps destined for the patient in the first place, /.../ which can also lead to unnecessary anxiety.

Ortho-2

Some physicians considered changing their use of the EMR to not writing about suspected serious diseases and instead wait with the documentation until the results are back, and the patient is informed.

I am more cautious about how I pronounce that; there could be misunderstanding /.../, they may not understand what it means. /…/ But we await the investigation and then when all the surveys and samples are back, then I would like to meet the patient first, inform, and then write that “now we have progression, the disease is back”.

Onco-1

Possible changes of writing practices were mainly discussed in relation to the medical jargon or suspected diagnoses and not, for example, in relation to possible offensive notes. Several physicians mentioned, that because the patient always had the right to request a copy, they would already write in a respectful manner. Only few doctors considered adapting their writing slightly, to make use of the system as a way to communicate with the patient directly (eg, include instructions for self-care or different types of reminders for the patient):

It is possible that I change a little bit in my way of writing; you can sneak in some messages there. I do not think it is something I avoid writing, but more that, if I know that the patient reads I might add a sentence here and there about things that I want to stress at the end, just for the patient, yes, quit smoking or something like that.

Onco-2

However, most physicians did not know, whether their patients read the medical record. Although some of them assumed that this is the case, they do not feel the need to ask the patients about this. One physician assessed that the patient portal had no impact on the medical work:

Yes, it is zero impact to be honest, because no one has been in contact and I feel that I am fairly clear when writing. So if someone has read his medical record then I have not heard about it, since they have in any case not been in touch and said that it is something that they do not understand or something is not right.

Ortho-3

### Process

This theme concerns requirements such as delayed access to and signing of records by physicians, as well as the question, who is the process driver (the patient or the physician). This involves also possible interferences and interruptions by patients, which is related to an increased stress for the physician due to time limitations and challenges on their workflow.

The physicians discussed an aspect that differed requesting paper copies of the record from accessing them over the Internet through the patient portal. To request the paper copy involved a delay that prevented patients to read the record before the physician.

Well, it happens of course very often [that patients request hard copies], but it is seldom that the patient receives any information that you do not already know about /.../ there is a ‘delay’ anyway, so we have a chance to catch up on the implications of the new findings and also think through a strategy that is also naturally then discussed with the patient at the same time.

Ortho-3

As described in the Background and Theory section, this delay was built-in into the patient portal by a “respite” when it was launched. Within the first 2 weeks, the patient could only access information that was signed by the physician. Only after a 2-week “respite,” unsigned records could be read by patients, which were then specially marked as unsigned. The time in which physicians signed the records differed, and there seems to be no standard practice. Several physicians reported during the interviews that patients had contacted and requested them to sign records, so that the patients can access them.

I got an email from a patient that she wanted to see the test results that I had not signed. I had not signed because it was an elevated test so I wanted to discuss with a colleague and see what we should do. I was told to sign because she wanted to know the answer, and she can not know what it means, she just sees an increased value, and I have to think about other investigations.

Onco-1

For some physicians, this increased their stress in that not only the records had to be signed on the patient’s demand but the physicians also had to “catch up” in terms of collaborating with colleagues and informing patients about results timely.

But now everyone will call, because they have seen the test results and want to know why, so they want a decision at once. So, yes, if all patients would go in and look at the test results, then this is great pressure for us to catch up, have time to write letters, call patients and not to take two-weeks until the next visit, but the day after.

Onco-1

Some can become calmer actually, when they have seen the test results, and know what they are. But not the oncologic patients, they are mostly worried.

Onco-1

When the “respite” was removed, patients were able to read the records regardless whether physicians had signed them or not. Hereby patients can read the test results immediately after they have been entered into the record, meaning that a patient might read the results before the physician. This was heavily criticized by the physicians, especially that patients could access the information before they had read it and come to a decision or finished their investigation.

I have a long education, and I do tests and other things and then I put all these things together and in the meantime, while I do the investigation, I do not want anyone to put their nose in this. And then I meet the patient and /.../ I think it can be very dangerous if the patient comes in during this investigation and sees the test results.

Onco-1

The patient should at least not be able to read before I have signed it! /.../ the final result might differ from the preliminary. It is not good if the patient is allowed to read the preliminary statement. They should only be able to read the final answer, otherwise it is not good.

EM-1

Patients reading the record before the physician had made sense of it and informed the patient was seen as a risk that could lead to undue anxiety among patients. This anxiety would then lead to patients calling their physician for answers at a time, when the physicians themselves may not have had the time to read up on the results and prepare the talk, or they might get interrupted while dealing with other patients.

The problem is that patients can come in and read information about themselves, and all findings, at a time when physicians often did not have time to read the same results. And even less have been able to discuss the matter and reason with the patient about it, or give notifications at that stage. Many times it requires that you consult with colleagues about the situation before you can come up with a proposal.

Onco-3

The examples given, related to the situation that patients read something before the physicians, were often rather drastic, for example, patients learning about an initial diagnosis of cancer through reading their medical record at home without a physician present. This example was not only given by physicians specialized in oncology but was indeed common also from other specialties such as orthopedics:

It is also important that we /.../ discuss the meaning of it. What do we do now, and what can this lead to as consequences and all this? That is nothing that you just bring up completely unprepared, but it often requires a lot of reflection. /.../ That the patient is informed about this by reading it at home, and perhaps with an incomprehensible language, it can be terrible anxiety driving.

Onco-3

I think it is the doctor’s task to interpret and filter it, to make it understandable /.../. It could also be that it is a sign of a serious disease, and it should not be told through the Internet as well, alone at home, so I think this is completely wrong. I mean, of course, the patient should have access to their medical records, but not in that way.

Onco-2

One of the oncologists emphasized repeatedly, that the particular medical specialties in health care have also different requirements:

We are aware of that [the trend of patient empowerment] and we are not opposed to that. But on the other hand, you need to understand that different businesses look different and the conditions, and it is not just about that some are surgeons and others are medicine people and some third are psychiatrists. But it is much more about how we handle the problems that occur and how we make the patient involved in it. And in oncology, we have been taught for decades that this is very important and we spent lots of energy on it, to handle this and then this comes along. Suddenly everything is just thrown overboard, for an entirely new situation that no one has really thought about.

Onco-3

However, one oncologist identified the opportunity that reading the record could increase patients’ participation and encourages them to read, while facing possible consequences:

It increases the participation in healthcare, and many patients can access their test results especially when they know that they will have a planned patient visit. And then I tell them that they should access it, but that they also need to take the consequences that they must wait until their next planned patient visit to discuss the results. And I believe that this suits some patients, but many patients also say that: “I do not dare”. And then to just read the notes about the disease contributes to improved engagements. So I am positive to this if it does not result in many problems or harm for the patients. For example, that they get notified at strange times when they cannot contact anyone. But when the patient is aware about this, then I do not see any problems with it. I mean you must take responsibility for your actions and if you want to log in and look for the test results at a certain time, then you have to take responsibility for it even if it is in the middle of the night.

Onco-2

### Workload

This theme concerns physicians’ views on the effect of the patient portal on their workload. The aforementioned anticipated need to change writing to be suitable for a layman was 1 aspect that physicians expected to add to their workload. It would not only make the record less efficient for communication with colleagues but would also take more time to write.

In addition, workload was presumed to be affected by requests to change something in the record and an increase of the number of patients’ questions. In the interviews, the latter was connected to assumed lack of medical knowledge by patients, which would increase their demand for explanations. Physicians also expected that when a patient reads, the focus might be put on details (eg, laboratory results) without seeing the overall picture. This could then dominate the discussion, where the physicians would have to explain in more detail why a raised value in this case is not that relevant.

Well, do they understand them then? The sedimentation rate was 23, but the normal is 20, and you have 23, then no one needs to bother about that. /.../ They are not able to interpret the test results, and it leads to more work, revisits and telephone calls, and they are worried. /.../ A thing like this is nothing but a lot of extra work /.../. I see nothing positive in that the patients read their medical records online.

Ortho-1

Connected to this is also the anticipated need of having to explain, and to convince the patient, what role a specific piece of information should play for the full picture or what treatments are possible alternatives. Physicians envisioned that the patient might use test results to search for alternative treatments in the Internet that might not be appropriate for the specific patient’s case or is not a treatment that is used in Sweden.

One can speed up the investigations and that is for the patient’s sake, and they can be prepared for what they will hear at the medical meeting. So it may be a good thing with it that they are prepared. They have seen the test results. They know roughly what it could mean and focus on the opportunities available as treatments. They also read about what options they have before they come here; they are well-read in that case. And then there will be greater demands when they come. And it is not certain that they have understood what they have read, but they just saw something and are stuck on what they think is best for them /.../ so it takes time to explain why we do not choose such a treatment.

Ortho-1

However, some physicians pointed out, that today patients do not understand everything in conversations with the medical professionals either and viewed the patient portal as a good opportunity:

The opportunity is that they are informed in another way and that is good in itself. Because I do not believe that patients understand all times what one says and they can then read it in the record so that they understand. Then it is good.

IM-2

The opportunity is that they are informed in another way and that is good in itself. Because I do not believe that patients understand all times what one says and they can then read it in the record so that they understand. Then it is good.

EM-1

I think we are quite bad at giving patients /.../ recommended treatment and plans and those type of things for the future. /.../ Because we say a lot of things /.../ and you know that when the patient returns home he or she will have forgotten 75% of what we have told them. But if we have put it into the record and patients can go into it and read what we actually said, this must be a good thing.

IM-2

Some physicians mentioned that patients are better read up today and perceived it as rather positive, that patients may use the record to look for further information in libraries or the Internet to learn more about their condition.

Patients can be very informed, and they come with the papers and they can be very well prepared ... and with relatives, too, so then you discuss different things.

Onco-3

Some physicians discussed the possibility that workload could remain unchanged or decrease because of patient’s possibility for self-service and self-management:

Patients have greater insight opportunity and can potentially be given greater responsibility for their own monitoring/follow-up care.

EM-2

We do not know quite yet, it could generate some phone calls but it could also take away some phone calls so therefore /.../ I do not think there will be an increased burden on the clinic, which is what many of my colleagues are afraid of.

Onco-2

However, the physicians related a risk of an increased workload not only to the contact with a patient but also to introduction of IT in health care on a more general level. Here, they transferred their previous (negative) experiences from other IT systems in health care, which had increased their workload:

It is very easy that the workload increases, and eventually one becomes more occupied with the systems than with personal meetings. If we take for example, Cytodos and what it takes for a single chemotherapy treatment. It takes a minute to fill out a paper form, but in Cytodos it may take five, ten minutes, so it is a factor of five times, and it can be up to ten to one, at some chemotherapy treatments.

Onco-2

In relation to expected risks of increased workload, it was also mentioned in numerous interviews that the work situation is already strained, with a lack of resources and also abounding administrative tasks. Many physicians expressed that there is a need for more staff in health care, to meet and interact with patients.

Patients want to e-mail and such things, and I think, it is a great way to communicate. The only problem is that there must be a structure for us /.../ so that it works, so that patients get answers /.../. I think, it is a matter of time and also a question of resources very much. If you want the patients to have more contact with us, get more information, and want us to more directly interact with patients /.../ then we need more people, basically.

Onco-3

### Control

This theme concerns the physicians’ worries that the patient portal might lead to patients monitoring and controlling the physicians. This is related to the possibility for patients to read their record as such, but often they refer to the log list, which enables patients to see who accessed their medical record.

I see no point in this! Is the patient supposed to act as the police?

EM-1

Most of the physicians explicitly state that they read records due to professional and medical reasons not for fun or entertainment.

The majority of medical personnel who are inside and read a record have no interest in doing it in some kind of aim to crave for sensational news. It is our job to make it as good as possible for the patient and therefore read or write in the record. It is not exciting like reading someone’s diary.

Ortho-2

The log list has evoked a feeling of distrust in numerous physicians, in that patients are suspicious, mistrust them, and want to guard their information from outsiders. In interviews, it was emphasized that the physicians have a long education and professional experience and that patients should be able to trust them.

This is going completely the wrong way! You become a servant from having been the expert who people asked. Now you are a servant that needs to be controlled, because you do not do your best.

Ortho-1

We speak the truth and now it feels that someone wants to watch us all the time! But we try to do our best! We do not work against patients.

Onco-1

In addition, the recorded logs could lead to misinterpretations, for example, when physicians read a patient’s record in a consultation in which they might not meet the patient personally (eg, in the role of doctor on duty, or when a colleague asks for a consultation) or if they accidentally logged into a record.

You can end up in pretty many records without having done anything wrong, but it can look like you have done something wrong.

IM-2

However, some of the physicians expect patients to read their records to ensure that the physician did not write anything by accident, as, for example, including wrong facts. Others saw the log list functionality as a way to reduce the risk of unauthorized access:

The advantage is that it is not possible to read someone else’s medical records without leaving a trace, which of course hopefully removes any pure curiosity medical record readers, like a neighbor or a relative. I think that is a much less of a problem than people might think.

Ortho-3

## Discussion

The thematic analysis revealed 4 themes that can be regarded as formative aspects of physicians’ TFs. The themes incorporate perceptions of the medical records as their work tool, the repurposing of which would have negative effects on their current processes and increase the workload. Certain aspects of the patient portal are seen as a threat in that it may be used for monitoring the physicians’ work. The physicians show concerns about their patients, who are seen as lacking medical expertise and might get harmed by using the patient portal.

### Work Tool

One interpretation of the physicians’ view of the record as their work tool is that they see themselves as its owners. They write the content, the information they add to the record is used by them to communicate with other health care professionals, to make a diagnosis, to form decisions, and to select a proper treatment. The transformation of the medical record to a patient portal is seen as time consuming and a threat to the effectiveness of their work tool. Some physicians also expect an upcoming need to change their way of writing, which was experienced as a negative and unnecessary effect on the work environment. In this, they identified possible requirements by the patients reading the notes, which is seen as repurposing the medical record, as discussed also in [13].

The record as such could also be repurposed in that it may serve as a communication tool between physician and patient, which could increase office efficiency as discussed in [3]. However, this transfer was not yet made by most of the physicians, who maintained their current frame of the record as their own work tool. With 1 exception, none of the physicians asked their patients whether they read records nor encouraged them to read. As limited staff engagement is 1 characteristic of low patient portal adoption [20], the lack of encouragement might not contribute to reach the objective of increased patient participation. However, Irizarry et al view the adoption by patients and the endorsement by medical professionals as a natural consequence, *“when existing patient portal features align with patients’ and providers’ information needs and functionality”* [21].

### Process

Before the patient portal, patients could request and read their records on paper. This was not seen as a controversial issue. One interpretation is that the physicians thought of this as a complex and time-consuming process that probably only few patients would undertake for reasons such as insurance claims and so forth. Another interpretation is the aspect of timing. Requesting and accessing the paper copy produces a delay, during which physicians can “catch up”: correct records that might have been wrongly dictated or other errors, do all diagnosing, and consult colleagues, and so forth. At the end of this decision-making process, patients were informed by the physician and presented with possible treatment options, and by then, patients would also receive the printout by regular post. The patient portal affected this timing, in that the possibility for a patient to read the record over the Internet removes the previous delay. The roles between patient and physician may potentially be changed.

Physicians refer to themselves as the ones responsible for the caretaking of patients. The physician should make sense of possible test results, make a diagnosis, consult colleagues if needed, and come up with a decision regarding treatment. The interviewed physicians generally preferred to complete all steps before giving information to and having a discussion with the patient. Physicians are trained to deal with medical issues, and with this mind-set, in addition to their view on patients’ difficulties to understand the content in the record, they are concerned that reading the record could harm the patient. In relation to this, only few physicians considered patients with chronic conditions who might be quite knowledgeable and therefore likely be able to understand the content and make use of it. As stated previously, patients reading the record before the physician does, was seen as a risk that could lead to undue anxiety among patients. Anxiety itself was judged to be harmful to the patient. It could also lead to other consequences, such as patients taking the wrong action on their own initiative (ie, ending a medication in advance). The interpretation is that the physicians see the risk of being unable to guide patients and their reactions in exceptional situations.

Web-based access does not only change the process with regard to the information flow, in that patients look up the information before the physician, it might also demand a change that starts well before that. By the time that the tests are taken, the physician might have to inform the patient about possible results, so that patients are “prepared” for what might end up in the medical record. Hence, based on this preparation, patients can decide for themselves, if they want to log-in to the record and accordingly will take the responsibility for it, if the information is bad. This change in the health care process can be seen as taking 1 step closer to patient participation and shared decision making, which is reflected in the phrase “nothing about me, without me” [22]. However, this clashes with the framing most physicians in this study have of their process and the extent to which patients can be included. In addition, a test may reveal an illness that was not anticipated by the physician. In this case, the patient could be caught off guard, although they had been sufficiently informed.

The physicians described, that patients demanding them to sign or asking for explanations at once would interfere with their work processes. A similar concern that patients may expect immediate responses to their requests was also stated in [3]. The physicians in this study perceived the process of gathering all relevant information and preparing themselves before informing the patient as the best way of caring for the patient, preventing undue anxiety. The analysis found “requests to sign” during this part of the process to be seen by the physicians as a negative consequence and an additional stressor for their work environment, especially since they had not been informed about changes made to their work processes. However, physicians might not be aware of the patients’ perceptions of this way of processing information. According to Rexhepi et al, the long period of waiting until the physician contacts them and only by this being able to receive the results was reported by patients to be worse than receiving bad results in the Internet by themselves [23].

Most physicians mentioned life-threatening diseases such as cancer when talking about the patient portal, not only the oncologists. The same narrative occurs in numerous interviews and could be seen as a social phenomenon in that the physicians as a group may have collectively constructed or shaped each other’s attitudes [15]. One interpretation is that this narrative about patients receiving the initial cancer diagnosis could be a repetition and reproduction of what the doctors read about in the media at the time of the study. Another interpretation could be that the physicians framed this particular group of patients as the most vulnerable. Most physicians viewed the patients as not knowledgeable when it comes to medicine or medical language and therefore expected them to become anxious through reading the hence incomprehensible record. Interestingly, no physician talked about chronically ill patients, who already do a lot of self-management, for example, diabetes [24]. Having a chronic disease often includes regular laboratory tests to be carried out, the results of which the patients often are able to monitor themselves, and where an appointment is only necessary if the results are out of range.

In addition, what is not discussed in these interviews is the possibility that patients can educate themselves regarding their disease, which can be facilitated when they read their records in a timely manner. Only 1 physician acknowledges that some patients know more about their diseases than the physician, especially if that is a rare disease and the physician has not read up before. This framing of stability of patients’ health literacy contradicts, for example, the view of health literacy as a key outcome from health education, which is seen as being critical to empowerment [25].

Although other studies have previously shown that health care professionals are concerned that patients might get worried or confused [3], this study could relate the concerns to a specific view of patients as vulnerable subjects, who do not understand the content of the record and may be harmed by reading it. This idea explains the physicians’ perceptions of the preferability of the current workflow, where the physician informs the patient.

### Workload

Numerous concerns related to a possible increase of workload were also based on the view that patients will not understand the content of the record. The physicians concluded that this would probably lead to: (1) increased phone calls, (2) longer discussions, and (3) a need to change the writing, which would take more time. However, the concerns about increased phone calls might be unfounded. For example, the cancer patients in the study by Rexhepi et al stated that they would wait for the next visit to ask questions instead of calling and demanding explanations at once [23].

The possibility that the patient portal could be a self-service tool, which possibly reduces workload, was not part of the framing of most physicians. This corroborates the results of [3], in which concerns regarding hampered workflow and increased stress outnumbered the view of potential office efficiency. Instead, the physicians in this study transferred negative experiences from other IT systems, which previously had increased their workload. In addition, it has been mentioned that administrative tasks have increased, leaving less time for the contact with patients. These experiences may have influenced their expectations regarding the patient portal.

### Control

Trust, as a vital part of the doctor–patient relationship, was an important aspect within this theme. Physicians referred on several occasions to their long education, in which they learned, for example, how to handle difficult cases and situations. Physicians had difficulty in seeing benefits of patients reading the records, and some physicians felt as if the patients wanted to control them. This is related to the work by Erlingsdottir and Lindholm [7] on professional autonomy of physicians and the encounter with patient portals. Some physicians indeed see patient portals as limiting their autonomy as professionals.

An interpretation of their framing of the patient portal as a surveillance tool is that due to the perceived lack of benefits for patients reading the medical content, what might be left to motivate patients to read is to monitor doctors’ activities in terms of: (1) who logged into the record and (2) whether the doctor entered the information correctly. The aforementioned study on cancer patients did show that patients felt more in control but rather in relation to their care and their own understanding of their health condition [23]. Only few patients mentioned an urge to read because of suspected incorrect entries, and those who found inaccuracies did not file a correction [23]. Furthermore, Huvila et al identified a diversity of patients’ positions toward reading their medical records and emphasize that it is important to take these into consideration and find flexible solutions instead of using rather simple demographic groups [26]. These positions were interrelated and did not include monitoring health care professionals as such but instead included the aspect of mistrust and the desire to control their health treatment.

In relation to the log list, most physicians felt the need to explain that there is no reason for them to read the records of *their patients* other than out of professional interest. However, the treating physician does not need to explain this, because from a legal perspective, being the treating physician is the justification for reading a medical record in the first place. Still, that patients read the log list was interpreted by the physicians as a possible mistrust toward the treating physician. An interpretation is that the physicians struggled to take the patient’s perspective, who might be concerned that potentially all employees in health care in the whole county can read their medical history, including, for example, neighbors, former life partners, and so forth.

In addition to the previous frames, to regard the patient portal as a tool to monitor the physicians’ work may increase the difficulty to identify potential benefits the work environment in health care, but rather stimulates resistance.

### Limitations

Limitations of this study include interviewing a number of physicians from different medical specialties where respondents were somewhat unevenly distributed among the fields. In interpretative research, it is however not critical to get a fully representative sample: to reach thick descriptions, which the study did, is more important. Although interviews are considered as a viable method to study TFs [14], a limitation of this study is that the analysis is restricted to the data that can be elicited in interview mode because no additional data could be collected (eg, through observations or surveys).

The interviews took place about 6 months after launch of the system, which could be criticized of being too soon as the participants did not yet know for certain how many patients made use of the patient portal or how it was used. However, we believe that the use of the concept of TFs is particularly useful in the early stage of deployment because the frames strongly influence the choices made regarding the use of the technologies [14]. The TFs may change over time, so results from this study may be useful as an important starting point to examine these changes. In addition, it would be interesting also to learn whether the physicians are today aware about whether their patients are reading their records. At the time of the interviews, most physicians stated that they neither know nor ask their patients, if they do.

Another limitation can be related to the frame domains. The collected material did not hold a sufficient content to make an in-depth analysis of the frame domain *technology strategy*, although particular questions were asked during the interviews. This can be explained by the fact that the physicians were not informed of managerial and political motives and argumentation behind the implementation.

### Conclusions

The analysis showed that the TFs of the physicians were significantly constructed based on assumptions and expectations and not experiences. This is not surprising due to the time of the interview taking place only few months after the launch. However, aspects such as patients’ rather stable lack of medical knowledge, demanding explanations, and increasing the workload influenced physicians’ overall assessment of the usefulness of the patient portal significantly. The constructed TFs of the physicians helped them to make sense of the patient portal by reducing some of the uncertainties. Hereby, the frames provided a basis for assessing its overall usefulness, as discussed in [14].

The transfer of a paper-based healthcare process, where patients read on paper, into a digital process challenges current work practices and has consequences for the work environment. Mostly, this is explained by the changing positions between the physicians and patients: the latter can drive the process, reducing the physicians’ ability to guide patients and their reactions in exceptional situations. Several physicians were concerned about having less time to read, consult colleagues, and to reflect.

The physicians’ experiences were expressed as worries: patients would not understand the content of the record, and they would become unnecessarily anxious from misunderstandings. However, some of the frames include assumptions and generalizations regarding patients and their abilities (ie, the patient as health illiterate). Albeit being accurate for some groups of patients, it disregards those patients who already actively participate in health care. This risk is in line with a study on medical students by Johnson et al, who conclude that medical teaching “must make students aware of the ingrained stereotypes that likely influence their perceptions of patients and that form barriers to accurate clinical assessment” [27]. Furthermore, a stereotypical view of patients as passive actors who lack medical knowledge and have to be protected can also significantly influence design decisions of health IT [28]. Thus, the reliance on frames that are based on stereotypes might inhibit a change in health care toward patient participation and influence the design of future IT systems. This study hence reveals a need to provide physicians with information about the values for patients from using patient portals.

Physicians drew attention to different processes and requirements regarding particular diseases and also in respect to different medical specialties, which might be important to take into account. This is in line with the concept of TFs, which includes context as a formative aspect of TFs [14]. However, patients’ experiences should be viewed on a more nuanced level. There is more to being a patient, for example, in oncology, than receiving the initial cancer diagnosis. It is also about dealing with a chronic disease over a long period on a day-to-day basis, where some patients are particularly experienced when it comes to their condition [29]. It is important to enable different stakeholders to express their view from their particular context, for example, the physicians in their particular medical specialty. However, from a patient-centered care perspective, another approach could be to shift focus from medical specialties toward patients, who might be treated by physicians from different specialties at the same time.

As the overarching political aim is to increase patient participation, it is crucial to inform and include physicians in the change process. Patient participation is not obtained through an introduction of a patient portal alone. One example of change is that the patient portal made it possible for patients to access information before physicians. This is changing a work practice and turning a current workflow upside down. It is not surprising that physicians do not welcome this imposed change, which is reflected in this study in their way of finding workarounds (eg, not signing the record; not writing suspicions). Hence, the overall objective of patient participation might not pervade the health care process, if it is not fully understood by physicians, and the technology supporting this goal is seen as a threat imposed top down. A change of work practices may be beneficial to increase patient participation, but such changes should preferably be designed and discussed with physicians. However, the strong resistance from the physicians made this challenging when launching the patient portal.

The results of this study may lead to an impression that all physicians shared a negative attitude toward the patient portal. However, although most of the interviewed physicians expressed concerns in one way or another, the strength of the concerns varied from a really strong to neutral to positive attitude. In addition, the questions during the interview explicitly aimed at exploring both positive and negative concerns. Differences in attitude could be explained by or influenced by: (1) length of work experience, (2) medical specialty, (3) gender, (4) physician’s personal experiences (eg, being a chronic patient himself or herself), (5) general attitude toward technology, and so forth. However, the present analysis did not reveal any solid patterns in this small sample.

Further research is needed to investigate the extent of substantiation of the expressed concerns and also how patients actually make use of the patient portal, that is, how they deal with inconclusive or incomprehensible information. Having a more comprehensive view on possible benefits for and current use of the portal by patients could help physicians to consolidate or adapt their TFs accordingly. This could also be a way to identify possible positive effects of the patient portal on the work environment and how medical professionals could profit from its potential. In addition, well-founded concerns should be addressed in the design of patient portals, together with patients’ benefits, that need to be strengthened and communicated to the medical profession.

## Appendix

Multimedia Appendix 1 Interview Template.

## Abbreviations

CAQDAS: Computer-Assisted Qualitative Data Analysis Software
EM: Emergency Medicine
EMR: electronic medical record
IM: Internal Medicine
IT: information technology
Onco: Oncology
Ortho: Orthopedics
TF: technological frames
