# Supplementary material for: “The Record is Our Work Tool!”—Physicians’ Framing of a Patient Portal in Sweden
Source: J Med Internet Res. 2016 Jun 27;18(6):e167. doi: 10.2196/jmir.5705 (PMC4940602; doi:10.2196/jmir.5705)
Supplement: Multimedia Appendix 1 [file jmir_v18i6e167_app1.pdf]

Multimedia Appendix 1 - Interview Template

Introductory questions

1. What medical speciality do you work in?
2. Number of years in practice.
3. Gender.
4. How much do you estimate that you use IT in your daily activities? (suggested answers in % of your work time)
5. What do you think about patient involvement in healthcare? What does it mean for you to increase the patient's participation?

General questions regarding eHealth services and Journalen

6. What does e-health services mean for you? Give some examples!
7. What is your experience of e-health services for citizens?
8. Have you heard of the e-service *Journalen*?
9. What do you think is important when e-health services are introduced in your county?
10. How often do you meet patients who have read their records?
11. How often do you meet patients who have read their *paper* records? Or the records online? Do you find any difference between them?

Online access - Specific Issues

12. What opportunities do you see when the patient has access to their medical records online? What are your concerns?
13. Have records online affected the meeting with patients?
14. Have records online influenced your approach on a general level?
15. Is the way you document in the medical record affected by the currently available online for the patients and if so how?
16. What is your opinion regarding that the log data is accessible to the patient? Are there pros / cons with this? Will/does it affect your work environment?
17. What do you think about the possibility of having "respite" (time to correct and verify) before the patient has access to his/her latest record entry? How do you regard this, compared to a service that gives the patient direct access?
18. What do you think about the possibility for a patient to block certain information in his/her medical record?

eHealth Services - National and regional

19. What do you know about the national e-health strategy and its Action Plan on e-health services?
20. What do you know about the county and regional strategies on e-health services?
21. According to the action plan of CeHis (*Center för eHälsa i samverkan*; Swedish Center for eHealth), 100% of the inhabitants in Sweden should in 2014 have the possibility to access parts of their medical records and in 2017 to access his/her record information from all care and diligence, through the patient portal "My eHealth". What is your opinion about that vision?
22. Which e-health services do you currently use in your own work situation? Positive / negative experiences?
23. What needs of e-health services will you / your profession have in the future? Why?
24. Which actors in the health sector do you believe are interested in e-health services and in what respect?
25. Which groups of healthcare do you believe are interested in e-health services and in what respect?
26. Who do you think will benefit the most from e-health services?
27. Is there anything further that you consider in the context of e-health services that you think that we have not addressed?
